# Tau Depletion in APP Transgenic Mice Attenuates Task-Related Hyperactivation of the Hippocampus and Differentially Influences Locomotor Activity and Spatial Memory

**DOI:** 10.3389/fnins.2018.00124

**Published:** 2018-03-01

**Authors:** Misato Yoshikawa, Yoshiyuki Soeda, Makoto Michikawa, Osborne F. X. Almeida, Akihiko Takashima

**Affiliations:** ^1^Department of Aging Neurobiology, National Center for Geriatrics and Gerontology, Obu, Japan; ^2^Department of Pharmacology, Shujitsu University, Okayama, Japan; ^3^Clinical Research Center, Fukushima Medical University, Fukushima, Japan; ^4^Department of Biochemistry, School of Medicine, Nagoya City University, Nagoya, Japan; ^5^Department of Stress Neurobiology and Neurogenetics, Max Planck Institute of Psychiatry, Munich, Germany; ^6^Laboratory for Alzheimer's Disease, Department of Life Science, Faculty of Science, Gakushuin University, Tokyo, Japan

**Keywords:** tau, amyloid beta-peptides, hippocampus, hyperexcitation, Dementia

## Abstract

Hippocampal hyperactivity, ascribed to amyloid β (Aβ)-induced imbalances in neural excitation and inhibition, is found in patients with mild cognitive impairment, a prodromal stage of Alzheimer's disease (AD). To better understand the relationship between hippocampal hyperactivity and the molecular triggers of behavioral impairments in AD, we used Mn-enhanced MRI (MEMRI) to assess neuronal activity after subjecting mice to a task requiring spatial learning and memory. Depletion of endogenous tau in an amyloid precursor protein (APP) transgenic (J20) mouse line was shown to ameliorate hippocampal hyperactivity in J20 animals, tau depletion failed to reverse memory deficits associated with APP/Aβ overproduction. On the other hand, deletion of tau alleviated the hyperlocomotion displayed by APP transgenics, suggesting that the functional effects of Aβ-tau interactions reflect the temporal appearance of these molecules in individual brain areas.

## Introduction

Gradual worsening of memory and eventual impairments of executive functions are the main clinical features of both sporadic and familial Alzheimer disease (AD). The neuropathological correlates of these phenotypic characteristics include extracellular deposits of amyloid β (Aβ) that eventually form senile plaques and intracellular hyperphosphorylated tau that aggregates into neurofibrillary tangles (NFTs). Both Aβ and tau pathology are thought to contribute to the massive neuronal atrophy seen in the AD brain (Crimins et al., 2013) and genes that result in Aβ overproduction display have 100% penetrance in affected individuals (Tanzi, 2012). Treatments aimed to reduce the generation or deposits of Aβ with β–and γ-secretase inhibitors or antibodies against Aβ were shown to restore memory in mutant mouse models of AD, albeit without reversing the neuronal loss usually associated with Aβ overproduction (Janus et al., 2000; Fukumoto et al., 2010; Netzer et al., 2010). Thus, Aβ disrupts mnemonic functions, apparently by interfering with the function of brain circuits (Palop and Mucke, 2010), e.g., the ability to induce synaptic long term potentiation (LTP) (Walsh et al., 2002). In an extension of those studies, Roberson et al. implicated tau protein in Aβ-induced memory impairment; these authors found an amelioration of memory deficits (and survival) when *amyloid precursor protein* transgenic (*APP* Tg) mice were cross-bred with *tau* knockout mice (Roberson et al., 2007). Moreover, tau deletion resulted in a recovery of LTP in *APP* Tg mice (Shipton et al., 2011). These preclinical findings are consistent with those of a recent cross-sectional study strongly suggested a causal link between NFT lesions, rather than Aβ deposits, with cognitive decline (Brier et al., 2016); the latter may explain why Aβ-targeted therapies have generally proven ineffective at halting disease progression in subjects with mild cognitive impairment (MCI), a precursor of early-to-moderate AD (Holmes et al., 2008).

An abundance of evidence suggests that Aβ and tau AD pathology appear to mutually drive each other (Jack et al., 2013; Musiek and Holtzman, 2015) and that neuronal activation may increase either the aggregation, deposition and/or propagation of Aβ and tau (Bakker et al., 2012; Wu et al., 2016). Nevertheless, understanding the specific roles and sites and mechanisms of action of these molecules is essential for progress toward the development of targeted AD therapies. Complementing previous studies in mice that described (epileptiform) hyperactivity of hippocampal neurons in *APP* Tg mice (Palop et al., 2007; Busche et al., 2012), aged human subjects with a significant Aβ burden, but who were nonetheless cognitively healthy, were found to display increased hippocampal activity, as measured by functional magnetic resonance imaging (fMRI) (Mormino et al., 2012). A more recent fMRI (cross-sectional) study in healthy older humans found an association between Aβ tau pathology, neuronal atrophy and aberrant hyperactivation of the hippocampus during memory encoding (Marks et al., 2017); subsequent mediation analysis of those data revealed causality between hippocampal tau and hyperactivation. In this study, manganese-enhanced MRI (MEMRI) was used to re-investigate the relationship between neuronal hyperactivation, APP overexpression, tau and memory performance in mice.

## Materials and methods

### Animals

Transgenic (J20) mice express human amyloid precursor protein (hAPP) with the Swedish (K670N, M671L) and Indiana (V717F) mutations under the control of the PDGF β-chain promoter (Mucke et al., 2000). J20 mice were crossed with a *tau*-deficient (*tau* KO) mouse (provided from Dr. Vitek, Duke University) (Dawson et al., 2001) to produce J20/tau KO mice. We used 8- to 13-month-old WT (*n* = 22), J20 (*n* = 19), tau KO (*n* = 17), and J20/tau KO (*n* = 26) mice in this study. All studies were approved by the local ethical board and complied with the guidelines for animal experimentation of the National Center for Geriatrics and Gerontology. Mice were kept in a 12 h light/dark cycle and had free access to food and water.

### Behavioral tests

*Exploration* of an open-field was assessed by placing mice in a clear Perspex cylinder (30 cm diameter) for 30 min. using video recordings. Spontaneous alternation was assessed in a Y-maze (40 cm arm lengths) and monitored with a CCD camera. Mice were placed at the end of one arm and allowed to freely explore the arms for 15 min. *Spontaneous alternation* (%), defined as a consecutive entry in any of three arms of the maze, was calculated using the formula: No. of alternations/(Σ no. of entries – 2) ^*^ 100. Real-time recorded images (sampled at 2 Hz) were evaluated using the public domain NHI Image J software (http://rsb.info.nih.gov/nih-image/).

*Spatial memory* was evaluated using the Morris water maze (MWM) (1 m diameter) test, as described previously (Kimura et al., 2007). Briefly, for hidden platform training, a platform (10 cm diameter) was placed 1 cm below the surface of the water (constant location in within-trials training, but randomly located in between-trials testing. Each mouse received 3 daily training sessions at 30 min intervals over 9 consecutive days. Mice that did not find the platform within 60 s were guided to it and allowed to stay on it for 10 s. For the probe test (10th day), mice had to swim for 60 s in the maze from which the platform was removed. An error score was calculated by measuring the total distance traveled to reach the quadrant in which the platform was anticipated.

Acquired images were analyzed using a customized Matlab-based software in conjunction with an image analysis tool box (Mathworks Co. Ltd.).

### Mn-enhanced MRI (MEMRI)

Body weight of mice was measured (Supplementary Figure 3). Mice received MnCl_2_ (20 mg/kg i.p.), returned to their home cages for 30 min before exposure to a novel environment (clear Perspex cylinder, 30 cm diameter) for a period of 2 h (cylinder moved every 30 min to prevent habituation), after which they were again returned to their home cages (90 min) before MRI scanning. Anesthesia was induced with 3.0% isoflurane/air and maintained with 0.5–1.5% isoflurane/air; throughout, deep core temperature and heart rate were monitored (SA Instruments, Inc., USA). Scanning was performed (4 h after MnCl_2_ injection) in a 4.7T AVANCE III PharmaScan (Bruker BioSpin, Germany). RF transmission and reception were applied with a 23 mm inner diameter birdcage volume coil. Images were acquired with 3D Fast Imaging using a Steady-State Free Precession (FISP) sequence [repetition time (TR) = 8 ms, echo time (TE) = 4 ms, flip angle = 20°, number of acquisition = 7, matrix = 160 × 160 × 160, field of view (FOV) = 20 × 20 × 20 mm, and voxel size = 0.125 × 0.125 × 0.125 mm]. The total acquisition time was 31 min. MRI data analysis was performed as described previously (Kimura et al., 2007), with the aid of a custom-developed Matlab function (2012a, MathWorks). Brain slices were aligned with reference to Bregma. MR images were realigned and registered non-rigidly to the mouse brain template constructed by aligning and averaging 10 subject images. All voxel data were smoothed using a 3-dimensional Gaussian filter (Matlab image processing tool box, version 5.02, Mathworks). Image intensities were normalized to the mean signal in the whole brain of each individual mouse. MR images were visualized with Osirix (version 5.0.2), an open-source software for navigating multidimensional DICOM images.

### Statistical analysis

All numerical data are presented as mean ± SEM. The significance of differences between two groups was assessed by Student's *t*-test, and differences between multiple groups were assessed using 1-way ANOVA, followed by Tukey's multiple comparisons test. Statistical analyses was performed using PRISM4 software (GraphPad Software Inc., La Jolla, CA). Differences were considered significant when *p* < 0.05.

## Results

### Optimization of MnCl_2_ dose

**Figure 1:**
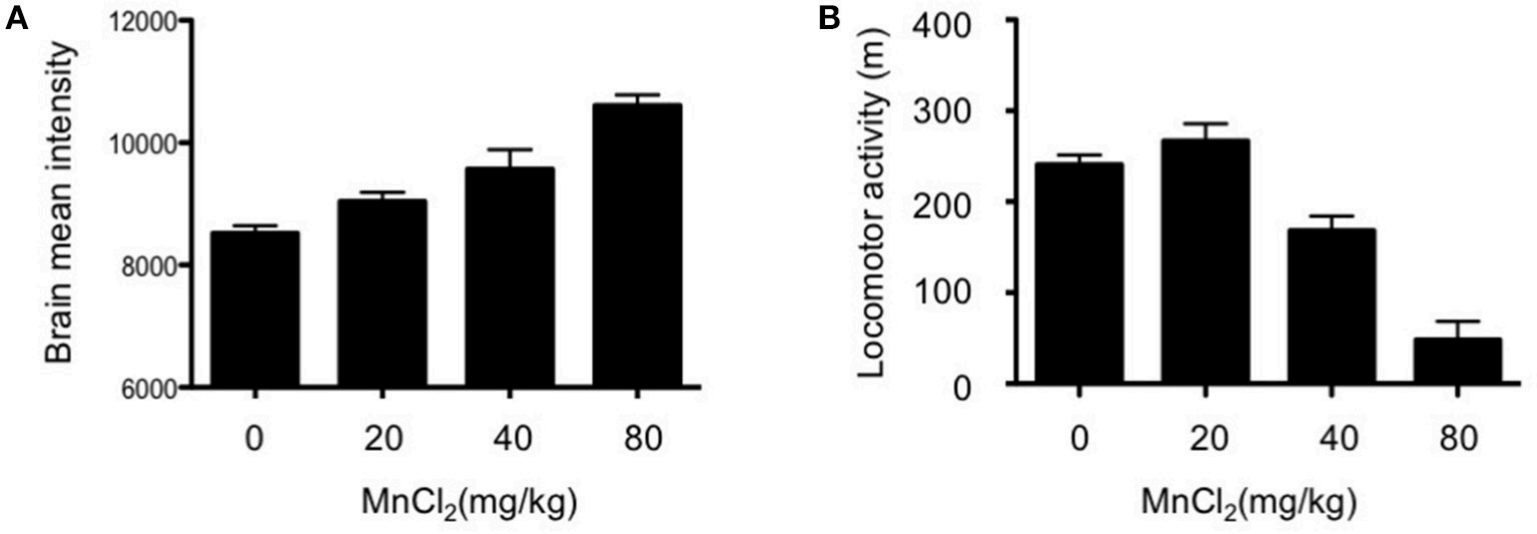
Dose-dependent increases in the mean intensity of MEMRI signal after application of MnCl_2_. **(A)** Mean signal intensity in whole brain 4 h after MnCl_2_ injections at doses indicated (0 and 20 mg/kg: *n* = 5/group; 40 and 80 mg/kg: *n* = 5/group). **(B)** Locomotor activity in C57BL/6 mice (5–8 months old), measured in an open-field for 30 min following i.p. injections with the indicated doses of MnCl_2_ (0 mg/kg: *n* = 6/group; 20, 40, and 80 mg/kg: *n* = 5/group).

MEMRI exploits the paramagnetic properties of manganese (Mn^2+^) to enhance tissue contrast of the MRI signal. Cellular uptake of the contrast agent, whose radius and chemical properties resemble those of calcium (Ca^2+^), is facilitated by Ca^2+^ channels; this allows it to be used as a reporter of neuronal activity (Silva and Bock, 2008). Although Mn^2+^ only poorly penetrates the blood-brain-barrier, excessive dosage can induce manganism with Parkinsonian-like symptoms (Sepúlveda et al., 2012) which present a potential confound in the interpretation of behavioral assays. Therefore, we initially determined the optimal dose of MnCl_2_ (i.p.) in wildtype (WT) mouse. As shown in Figure 1A, there was a dose-dependent increase in the mean intensity of MRI signal in mouse, with doses >20 mg/kg resulting in gradual decreases in locomotor activity Figure 1B. Accordingly, MnCl_2_ was administered at 20 mg/kg i.p. in all subsequent experiments.

### App overexpression is associated with hippocampal hyperactivity

**Figure 2:**
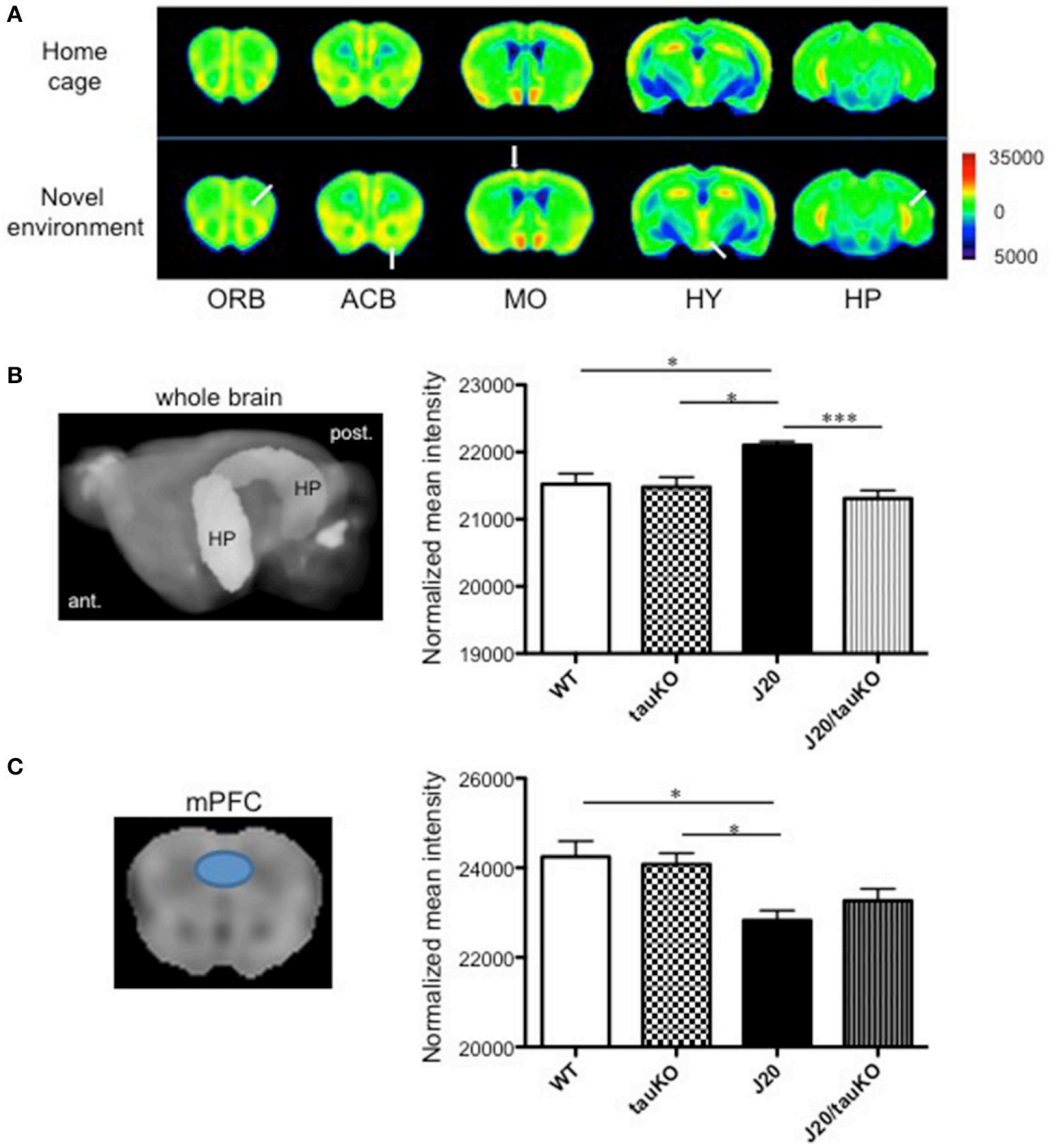
Differential brain area-specific and genotype-specific neural activation after place/contextual learning. **(A)** Shown are coronal brain sections from mice (C57BL/6 background) aged 5–8 months old, in their home cage (*upper*) or new (*lower*) environments. Relative MRI signal intensities, after normalization to mean signal intensity in the whole brain), are depicted (see color spectrum scale bar); in *lower* panel, white arrows indicate activation in regions of interest. In WT mice, neural activity in the orbito-frontal cortex (ORB), accumbens (ACB), motor cortex (MO), and hippocampus (HP) was enhanced after exposure to a new environment (*n* = 5) vs. home cage setting (*n* = 6). Place learning also increased activity in the hypothalamus (HY) of WT mice. **(B**,**C)** *left-hand panel* shows the hippocampus **(B)** and medial prefrontal cortex (mPFC) **(C)** as region of interest (based in Allen Mouse Brain Atlas) used to quantify MEMRI signal intensities to obtain the normalized data depicted in the *right-hand panel* where comparisons are made between WT (*n* = 6–7), J20 (*n* = 6–7), tau KO (*n* = 7), and J20/tau KO (*n* = 8) mice (aged 8–11 months) exposed to the place/contextual learning paradigm immediately before scanning. ^*^P < 0.05, ^***^P < 0.001.

Place learning (vs. home cage) produced strong MEMRI signals in the accumbens, motor cortex, and hippocampus of WT mice (Figure 2A). Compared to age-matched WT mice, APP-overexpressing J20 mice displayed increased hippocampal activity (2% normalized mean MEMRI intensity signal; *P* = 0.0134, Tukey's multiple comparison test) immediately after place learning (Figure 2B); on the other hand, the behavioral task did not alter signal intensities in the accumbens and motor cortex.

### Hippocampal hyperactivity, but not mPFC hypoactivity, is attenuated by deletion of tau

A pioneer study demonstrated that memory impairments in APP-overexpressing (J20) mice can be rescued by tau deletion; it was suggested that tau depletion results in an amelioration of APP-induced hyperactivity of hippocampal neurons and therefore, cognitive improvement (Roberson et al., 2007). In a replication of that study, we here cross-bred J20 with tau knockout mice and confirmed the original observation by MEMRI scanning of mice that had performed the place learning task: specifically, whereas MEMRI signal intensity in the hippocampus did not differ between WT and tau KO mice, and J20 mice displayed approximately 2% higher intensities than wildtype and tau KO mice, MEMRI signals were similar between J20/tau KO, tau KO, and WT mice (Figure 2B). In contrast, MEMRI signal intensity in medial prefrontal cortex (mPFC) of J20 mice was lower than that observed in WT and Tau KO mice, an effect that was not reversible by depletion of tau (Figure 2C). Thus, tau contributes to the increased neural activity in the hippocampus, but not the mPFC, in APP-overexpressing J20 mice.

### Behavioral correlates of hippocampal hyperactivity and its reversal by deletion of tau

**Figure 3:**
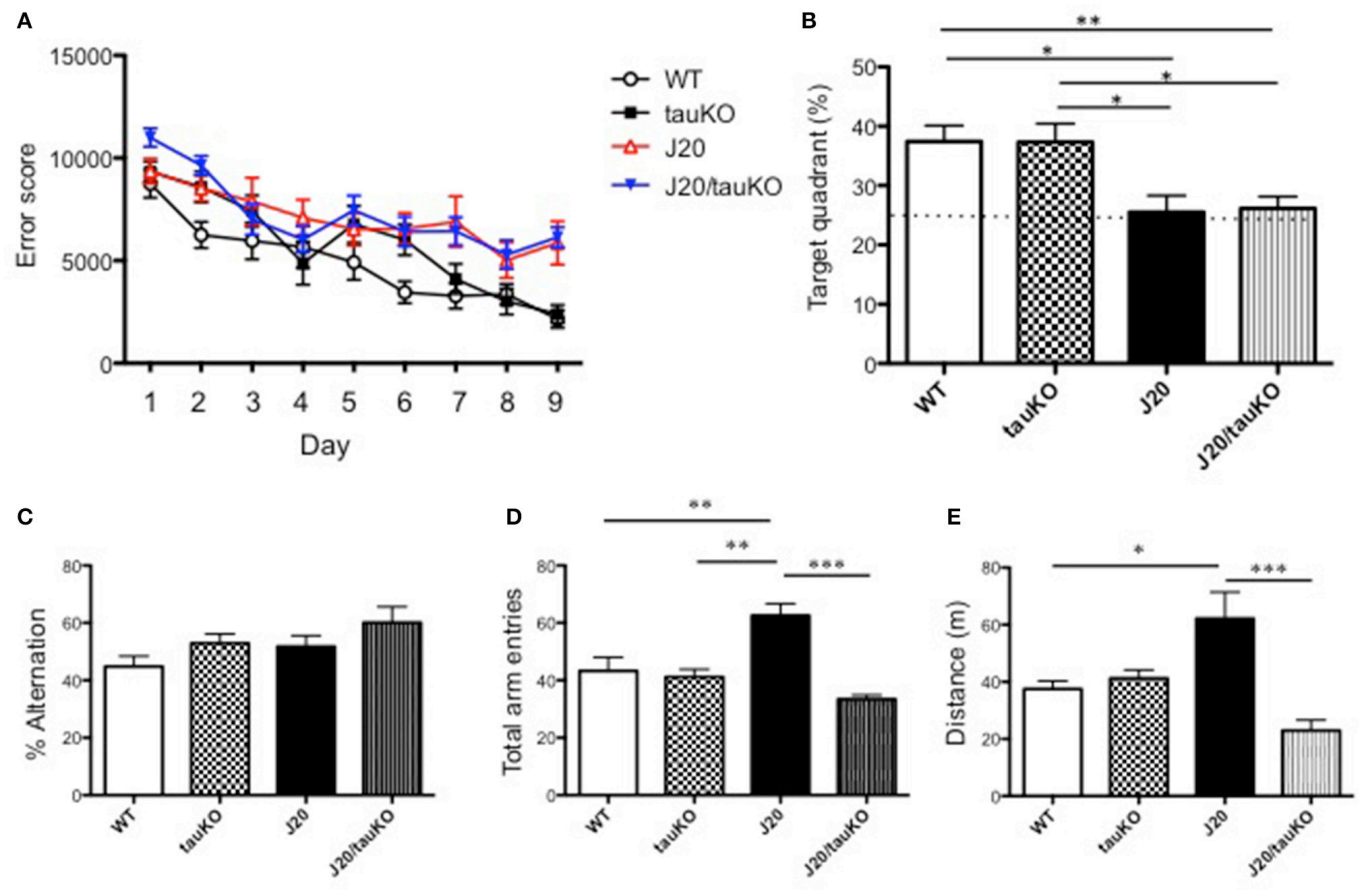
Depletion of tau reverses hyperlocomotor behavior but not place memory deficits in J20 mice. **(A)** Place memory was tested in the MWM in mice aged 8–12 months. Acquisition profiles, showing error scores during daily training trials (9 consecutive days) are shown. **(B)** Shows performance (time spent in target quadrant) in a probe test on day 10. Experimental groups in **(A,B)** were WT (*n* = 15), J20 (*n* = 12), tau KO (*n* = 10), and J20/tau KO (*n* = 18). In the Y-maze, alternations between the two arms of the maze (% time) (*P* = 0.4497, WT vs. tauKO; *P* = 0.6388, WT vs. J20; *P* = 0.0728, WT vs. J20/tauKO; *P* = 0.9969, tauKO vs. J20; *P* = 0.5895, tauKO vs. J20/tauKO; *P* = 0.5328, J20 vs. J20/tauKO). **(C)** Number of entries into the arms of the maze **(D)** were monitored in WT (*n* = 6), J20 (*n* = 5), tau KO (*n* = 7), and J20/tau KO (*n* = 5) mice (aged 10–13 months). Panel **(E)** shows distance in WT (*n* = 7), J20 (*n* = 7), tau KO (*n* = 7), and J20/tau KO (*n* = 6) mice during the first 5 min exploration of an open field arena. ^*^P < 0.05, ^**^P < 0.01, ^***^P < 0.001.

As mentioned above, Roberson et al. (2007) implicated tau in the parallel display of hippocampal hyperactivity and memory deficits by APP-overexpressing mice. Since those observations have not been corroborated thus far, we here investigated the role of tau (and its absence) on memory formation. To this end, WT, J20, tau KO, and J20/tau KO mice were trained to navigate to an invisible platform in a MWM. Error scores after 9 consecutive days of training were lower in all groups as compared to scores during the first learning session (Figure 3A). A probe test of memory revealed that whereas the WT and tau KO groups spent significantly >25% of the time in the target quadrant, both the J20 and J20/tau KO mice were markedly poor in locating the quadrant with the escape platform (Figure 3B). Thus, tau depletion failed to reverse the memory impairment induced by over-production of APP.

Next, we used the Y-maze test to investigate alternation behavior in the four genotypes. Although all mouse lines groups alternated between the two arms of the maze at similar rates, around 50% (Figure 3C), the APP-overexpressing J20 made significantly more arm entries (Figure 3D) than either the WT, tau KO or J20/tau KO lines. Together, these data suggested a role for tau in locomotor activity. This interpretation was supported by data showing that the locomotor activity of J20 mice in an open field arena is significantly higher than that of any of the other genotypes (WT, tau KO, and J20/tau KO) tested (Figure 3E). Together, this set of experiments indicates that, in J20 mice, the behavioral correlate of hippocampal hyperactivity is locomotor activity rather than place learning and memory.

## Discussion

Hippocampal hyperactivity is observed in patients with mild cognitive impairment (MCI) (Dickerson et al., 2005) but also in non-demented elderly subjects (Miller et al., 2008; Yassa et al., 2011). High levels of APP-derived amyloid β (Aβ) have long been linked to aberrant synaptic activity (Nitsch et al., 1993; Kamenetz et al., 2003) and strong associations have been established between Aβ, hippocampal activation and memory decline in otherwise cognitively-normal older humans (Mormino et al., 2012; Elman et al., 2014; Leal et al., 2017); accordingly, hippocampal hyperactivity may be a physiological accompaniment of aging in the mouse. Importantly, both cognitively-healthy aging humans (Sperling et al., 2003) and aged wildtype mice (Supplementary Figure 1) display reduced task-induced deactivation of the hippocampus. Interestingly, low doses of the anti-epileptic drug levetiracetam decrease hippocampal hyperactivity and improve memory in MCI patients (Bakker et al., 2012) and mouse models of AD (Palop et al., 2007); thus, memory deficits in AD patients and age-related cognitive decline are likely to be the consequence of shifts in the balance between excitation and inhibition of hippocampal neurons (see Busche and Konnerth, 2016).

Two recent positron emission tomography (PET) studies provide critical support that deposition of tau protein tracks and predicts cognitive decline during healthy aging (Marks et al., 2017) and in AD patients (Brier et al., 2016) better than Aβ. Tau, better known as a cytoskeletal protein, is now known to be located at synapses (Kimura et al., 2013; Kobayashi et al., 2017) where its potential to regulate neuroplastic events related to learning and memory has generated much interest. Clearly, tau pathology occurs downstream of Aβ (Jack et al., 2013) and a pioneering study by Roberson et al. (2007) implicated the essential role for tau in Aβ-associated memory deficits in the mouse. In this study, we confirmed that APP overexpression in J20 mice is accompanied by hippocampal hyperactivity; the latter was measured in mice aged 8 months old, an age at which Aβ deposition is seen in the hippocampus, prefrontal cortex and entorhinal cortex and when significant deficits in spatial memory are detectable in this mouse line (Mucke et al., 2000; Escribano et al., 2010; Wright et al., 2013). Further, we confirmed that tau contributes to hippocampal hyperactivity in J20 mice (cf. Roberson et al., 2007); briefly, J20 mice cross-bred to mice deficient in tau (tau KO) showed levels of hippocampal activity that were similar to those displayed by WT mice. Earlier authors proposed that the hyperexcitability observed in J20 mice results from Aβ -induced Fyn-mediated NMDA receptor activation (Ittner et al., 2010; Roberson et al., 2011) this interpretation is supported by reports that most APP transgenic mice exhibit spontaneous seizures and/or increased epileptiform EEG activity (Ziyatdinova et al., 2016).

Besides the aforementioned clinical studies, numerous studies in mice document a strong association between hAPP expression and impaired performance in the MWM (e.g., Palop et al., 2003; Galvan et al., 2006); the latter reports are supported by the demonstration that immunization against Aβ rescues learning and memory deficits in hAPP transgenic mice (Schenk et al., 1999; Janus et al., 2000) as well as the observation that hAPP mice display reduced synapse numbers and reduced inducibility of LTP (Shipton et al., 2011; Wright et al., 2013; Vega-Flores et al., 2014). Notably, our results do not conform with those of Roberson et al. (2011) who found that depletion of tau abrogates learning/memory impairments in hAPP mice tested in the MWM. As shown in Figure 2B, depletion of tau in J20 mice failed to reverse APP-induced memory impairments when the same test paradigm as that described by Roberson et al. (2011) was used; likewise, tau depletion did not improve cognitive performance by J20 mice in a variation of the MWM in which the escape platform is submerged (Supplementary Figure 2). While the discrepant findings on learning and memory performance in the MWM cannot be readily explained at present, it is worth noting that memory encoding and retrieval is regulated through reciprocal communication between the medial prefrontal cortex (mPFC) and hippocampus (see Eichenbaum, 2017) which, in turn, are likely modulated by dynamic spatio-temporal interactions between Aβ, tau and synaptic activity (see Leal et al., 2017; Marks et al., 2017). Since damage to the mPFC reportedly results in longer escape latencies in the MWM (de Bruin et al., 1997), the reduction in mPFC activity in J20 mice (Figure 2C) suggests that spatial recognition in the MWM test depends on both, hypoactivity in the mPFC and hyperactivity in the hippocampus. Inter-dependence between different brain areas (see Lalonde, 2002) and the potential for compensatory mechanisms is supported by the finding that there were no genotype-specific differences in alternation behavior.

Whereas tau depletion did not reverse the impaired memory phenotype of J20 mice, the hyperlocomotor behavior displayed by J20 mice was significantly reduced in the absence of tau. We recently reported that glutamate receptor stimulation induces AMPA/NMDA receptor- and GSK-3β-dependent local translation of tau in the somatodendritic compartment (Kobayashi et al., 2017). Since glutamate is the major fast excitatory transmitter in the brain, and because Aβ increases transmitter release (Abramov et al., 2009), it is plausible that Aβ enhances synaptic tau levels in the hippocampus and thus, hyperexcitation. Importantly, mPFC hypoexcitation in the J20 mouse does not appear to be tau-dependent, suggesting that Aβ modifies neuronal excitability and behavioral outputs through tau-dependent (hippocampus) and tau-independent (mPFC) mechanisms.

## Ethics statement

This study was carried out in accordance with the recommendations of NCGG of guidelines, NCGG animal experiment committee. The protocol was approved by the NCGG animal experiment committee.

## Author contributions

The study was conceptualized and designed by MY, YS, and AT; MY and YS performed experiments and analyzed the data; MM provided experimental animals; MY, OA, and AT interpreted the results; MY, YS, OA, and AT wrote the manuscript. All authors read and approved the manuscript.

### Conflict of interest statement

The authors declare that the research was conducted in the absence of any commercial or financial relationships that could be construed as a potential conflict of interest.

## Supplementary material

Supplementary Figure 1 Hippocampal hyperactivity is accentuated during aging. Wildtype mice (C57BL/6 strain), aged 10 (*n* = 5) or 20–23 (*n* = 6) months were scanned using MEMRI following placement in a novel environment. Note the reduced ability of the older group to display task-induced deactivation of the hippocampus. ^**^*P* < 0.01.

Supplementary Figure 2 Tau depletion does not improve performance in MWM with invisible escape platform. Following training and testing (3 d) with a visible escape platform, WT (*n* = 9), J20 (*n* = 9), tau KO (*n* = 7), and J20/tau KO (*n* = 8) mice (aged 7–10 months) were then exposed to 5 daily sessions during which the platform was submerged. **(A)** Acquisition profiles showing error scores during training trials (visible and submerged platforms). **(B)** Probe test results depicting performance on the 4th session with a hidden platform. ^*^*P* < 0.05.

Supplementary Figure 3 Tau depletion elevates body weight. Body weight was measured in WT (*n* = 7), J20 (*n* = 8), tau KO (*n* = 7), and J20/tau KO (*n* = 8) mice (aged 8–11 months) before MEMRI.
