# Supplementary figures and images for: Tau Depletion in APP Transgenic Mice Attenuates Task-Related Hyperactivation of the Hippocampus and Differentially Influences Locomotor Activity and Spatial Memory
Source: Front Neurosci. 2018 Mar 1;12:124. doi: 10.3389/fnins.2018.00124 (PMC5838015; doi:10.3389/fnins.2018.00124)

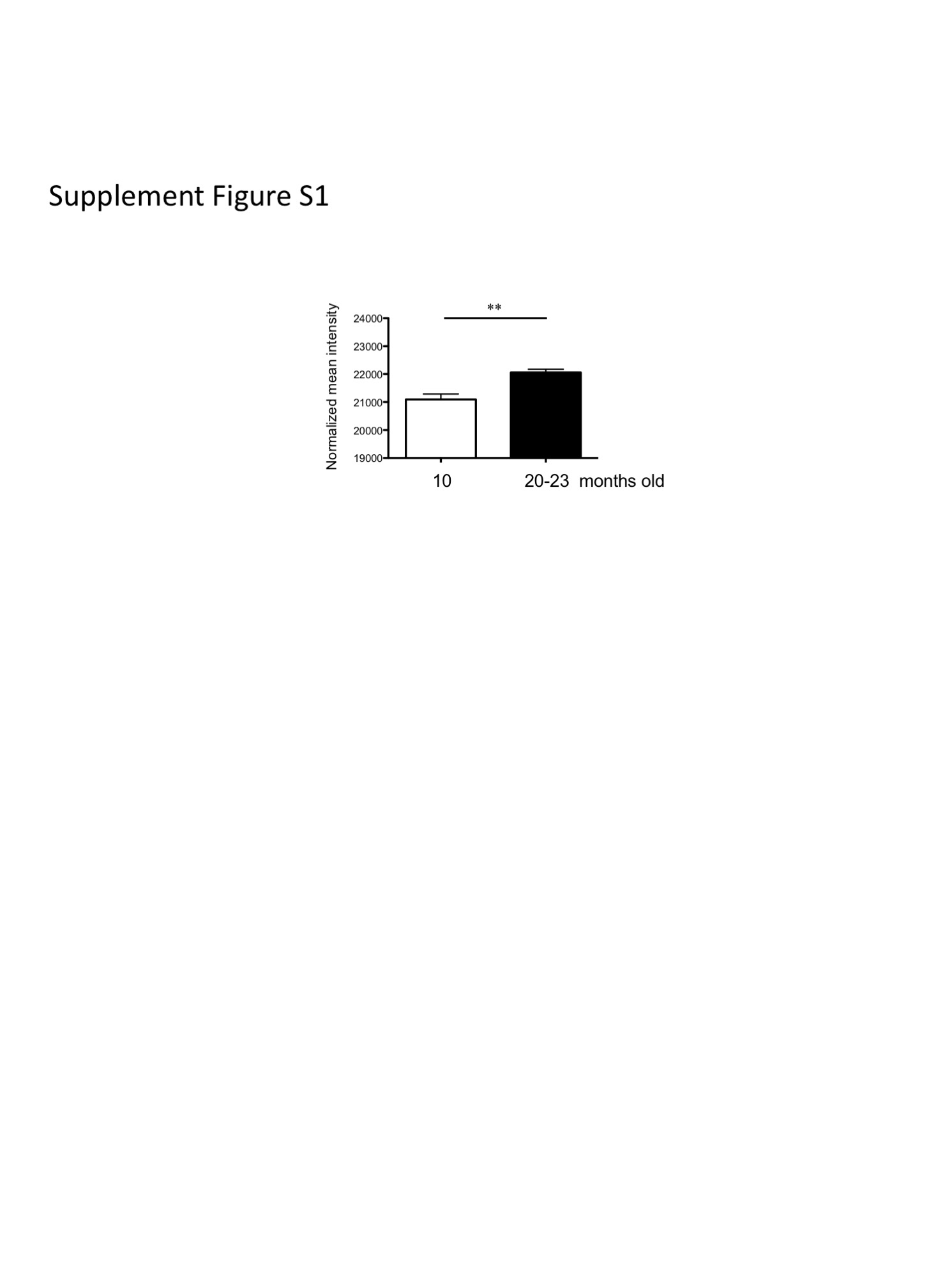

Supplement: Supplementary Figure 1 — Hippocampal hyperactivity is accentuated during aging. Wildtype mice (C57BL/6 strain), aged 10 (n = 5) or 20–23 (n = 6) months were scanned using MEMRI following placement in a novel environment. Note the reduced ability of the older group to display task-induced deactivation of the hippocampus. **P < 0.01. [file Image1.JPEG]

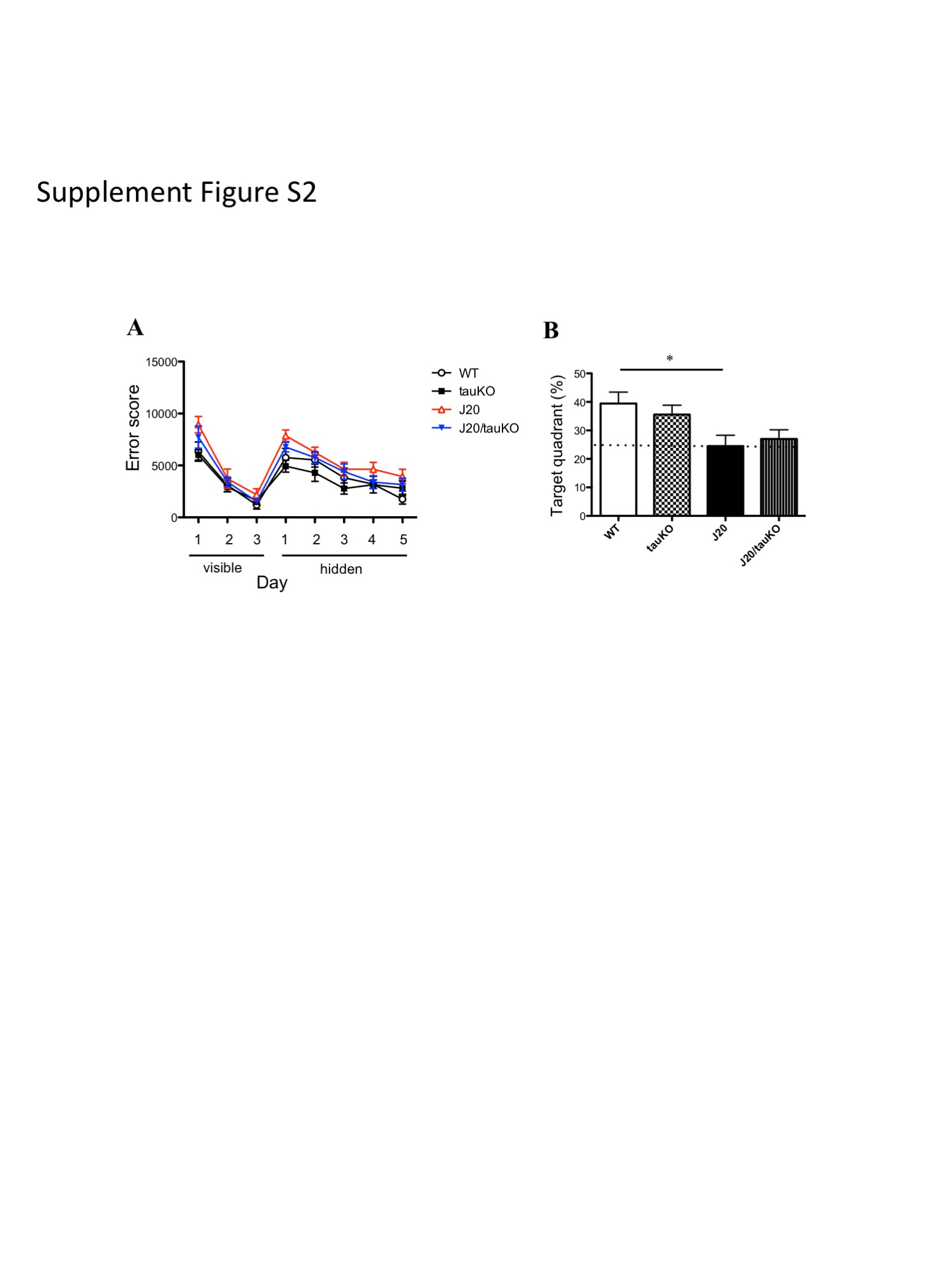

Supplement: Supplementary Figure 2 — Tau depletion does not improve performance in MWM with invisible escape platform. Following training and testing (3 d) with a visible escape platform, WT (n = 9), J20 (n = 9), tau KO (n = 7), and J20/tau KO (n = 8) mice (aged 7–10 months) were then exposed to 5 daily sessions during which the platform was submerged. (A) Acquisition profiles showing error scores during training trials (visible and submerged platforms). (B) Probe test results depicting performance on the 4th session with a hidden platform. *P < 0.05. [file Image2.JPEG]

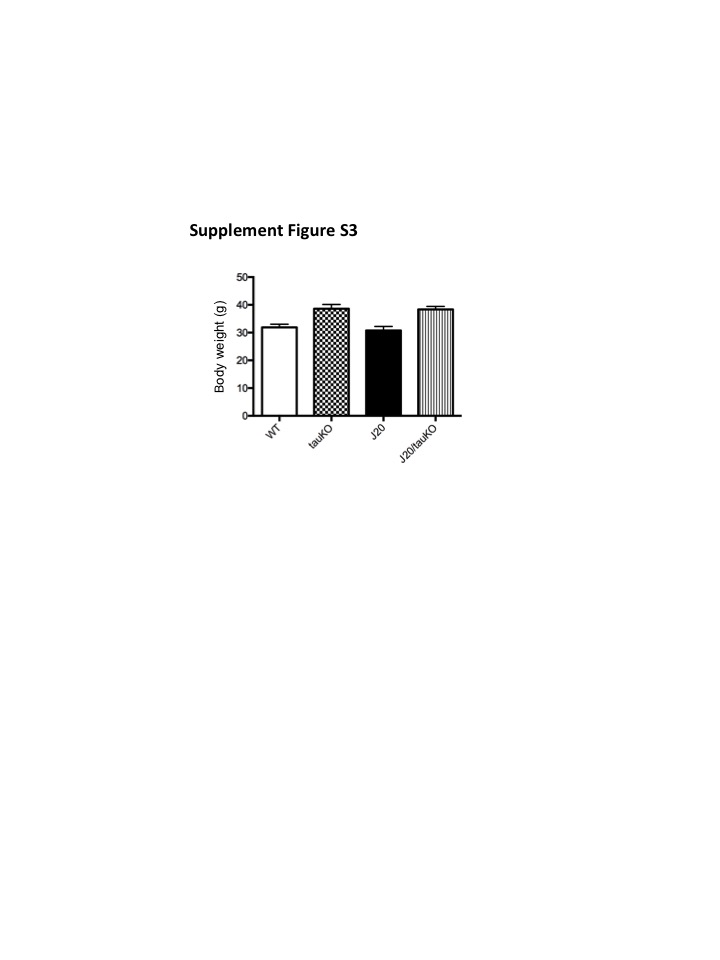

Supplement: Supplementary Figure 3 — Tau depletion elevates body weight. Body weight was measured in WT (n = 7), J20 (n = 8), tau KO (n = 7), and J20/tau KO (n = 8) mice (aged 8–11 months) before MEMRI. [file Image3.JPEG]
